# Genome-wide association mapping of quantitative resistance to sudden death syndrome in soybean

**DOI:** 10.1186/1471-2164-15-809

**Published:** 2014-09-23

**Authors:** Zixiang Wen, Ruijuan Tan, Jiazheng Yuan, Carmille Bales, Wenyan Du, Shichen Zhang, Martin I Chilvers, Cathy Schmidt, Qijian Song, Perry B Cregan, Dechun Wang

**Affiliations:** Department of Plant, Soil and Microbial Sciences, Michigan State University, 1066 Bogue St., Rm. A384-E, East Lansing, MI 48824 USA; Agronomy Research Center, Southern Illinois University Carbondale, Carbondale, Illinois 62903-7002 USA; USDA, Agricultural Research Service, Soybean Genomics and Improvement Laboratory, Beltsville, Maryland 20705 USA

**Keywords:** Genome wide association mapping, *Glycine max*, *Fusarium virguliforme*, SNPs, Quantitative trait loci mapping

## Abstract

**Background:**

Sudden death syndrome (SDS) is a serious threat to soybean production that can be managed with host plant resistance. To dissect the genetic architecture of quantitative resistance to the disease in soybean, two independent association panels of elite soybean cultivars, consisting of 392 and 300 unique accessions, respectively, were evaluated for SDS resistance in multiple environments and years. The two association panels were genotyped with 52,041 and 5,361 single nucleotide polymorphisms (SNPs), respectively. Genome-wide association mapping was carried out using a mixed linear model that accounted for population structure and cryptic relatedness.

**Result:**

A total of 20 loci underlying SDS resistance were identified in the two independent studies, including 7 loci localized in previously mapped QTL intervals and 13 novel loci. One strong peak of association on chromosome 18, associated with all disease assessment criteria across the two panels, spanned a physical region of 1.2 Mb around a previously cloned SDS resistance gene (*GmRLK18-1*) in locus *Rfs2*. An additional variant independently associated with SDS resistance was also found in this genomic region. Other peaks were within, or close to, sequences annotated as homologous to genes previously shown to be involved in plant disease resistance. The identified loci explained an average of 54.5% of the phenotypic variance measured by different disease assessment criteria.

**Conclusions:**

This study identified multiple novel loci and refined the map locations of known loci related to SDS resistance. These insights into the genetic basis of SDS resistance can now be used to further enhance durable resistance to SDS in soybean. Additionally, the associations identified here provide a basis for further efforts to pinpoint causal variants and to clarify how the implicated genes affect SDS resistance in soybean.

**Electronic supplementary material:**

The online version of this article (doi:10.1186/1471-2164-15-809) contains supplementary material, which is available to authorized users.

## Background

Sudden death syndrome (SDS) of soybean [*Glycine max* (L.) Merr.], caused by the soil-borne fungal pathogen *Fusarium virguliforme* [1], is a considerable threat to soybean production [2]. The fungus infects soybean root systems and produces toxins that are translocated to the leaves, resulting in premature defoliation and pod abortion [3, 4]. In recent years, SDS ranked among the top five most damaging diseases of soybean in the United States [5]. In the Midwestern soybean producing area of the U.S., it is estimated that SDS has resulted in average losses valued at $190 million a year [6].

Host plant resistance is believed to be the most effective control measure for SDS [7]. Since no soybean genotypes confer complete immunity to this disease, soybean breeders still rely on quantitative resistance to SDS [7, 8]. The wide range of variation of susceptibility to both leaf scorch and root rot also provides a great opportunity to improve SDS resistance through genetic manipulation [9]. However, most of what we know about the genetic architecture of SDS resistance is based on traditional quantitative trait locus (QTL) linkage mapping using bi-parental populations. Fourteen QTLs dispersed throughout the genome, underlying resistance to root infection, leaf scorch or both, have been confirmed in several bi-parental populations [10]. However, the large confidence intervals for those QTLs impair the precise identification of causative genes. To date, only one resistance gene (*GmRLK18-1*) has been tagged and cloned [11]. This gene is at 1.071 kbp on chromosome 18, within the major resistance QTL (*Rfs2*). Association mapping, which exploits historical recombination events at the population level, has become a powerful alternative to linkage mapping in the dissection of complex trait variation at the sequence level [12]. A more specific strategy, genome-wide association (GWA) mapping, is a powerful complementary strategy for classical bi-parental linkage mapping to dissect complex traits and has been used with success in *Arabidopsis* [13], rice [14] and maize [15, 16]. The use of association mapping in soybean was therefore desirable to improve the mapping of important traits in soybean. So far, only a few association mapping studies, with limited numbers of markers, have been reported for dissecting agronomic traits in soybean [17, 18]. To the best of our knowledge, GWA mapping has not yet been employed to study any traits related to soybean disease resistance. Recently, the availability of a soybean reference genome sequence and the development of high throughput SNP assays has enabled GWA mapping in soybean [19, 20]. A previous study reported that approximately 1% of the 6,037 Plant Introductions (PIs) from the USDA Soybean Germplasm Collection were partially resistant to SDS [21]. Therefore, conducting an association study in assembled PI collections might not be feasible. Furthermore, previous research indicated that most of the PIs showing resistance to soybean cyst nematode (SCN) were also partially resistant to SDS [22]. Therefore, association mapping with released elite cultivars is more likely to identify superior resistance alleles that have been captured and accumulated by SCN or SDS breeding practices.

The goal of this study was to investigate the genetic architecture of soybean SDS resistance in released elite soybean cultivars. Here we present the first experimental results of GWA mapping for SDS, across two independent panels of elite soybean cultivars, using a high-density customized oligonucleotide genotyping array. We detected 20 QTLs including known candidate genes (or QTLs) as well as new candidate loci in the soybean genome. The identification of these loci will increase our understanding of mechanisms underlying SDS resistance, and provide valuable markers for breeding soybean lines with SDS resistance.

## Methods

### Sampling and genotyping

Two independent experiments were conducted in this study. Experiment 1 was done with a mapping population of 392 diverse soybean cultivars (association panel P1), consisting of 251 varieties released between 2010 and 2012 and 141 advanced breeding lines from Michigan State University. Experiment 2 used a set of 300 diverse *G. max* advanced breeding lines (association panel P2) developed by public breeders. The germplasm was chosen to represent a range of materials developed for the U.S. North Central soybean production area. Further information about the P1 and P2 panels is given in Additional file 1.

Soybean genomic DNA was extracted from young leaf tissue following the previously described method [23]. All the accessions in panel P1 were genotyped using the Illumina SoySNP50k iSelect BeadChip (Illumina, San Diego, Calif. USA) which consists of 52,041 SNPs [20]. All the accessions in panel P2 were genotyped using the Illumina SoySNP6k iSelect BeadChip (Illumina, San Diego, Calif. USA), which consists of 5,361 SNPs [24]. The chromosomal distributions of the SNPs of SoySNP50K and SoySNP6k BeadChip are shown in Additional file 2. Genotypes were called using the program GenomeStudio (Illumina, San Diego, Calif. USA). The SNP data were coded according to the standard codes for nucleotides derived from the International Union of Pure and Applied Chemistry (IUPAC). The quality of each SNP was checked manually as previously reported [25]. SNPs without physical position information and with low quality (call rate < 80% and or minor allele frequency < 0.05) across all samples were removed from the dataset.

### Field resistance evaluation

The association panel P1 was evaluated for SDS resistance in a naturally infested SDS disease nursery at Decatur, Michigan during the growing season (May-October) in 2011 and 2012, where consistent, natural and heavy SDS disease symptoms was observed on susceptible checks. Four replications per year were grown in a lattice design with four-row plots 6 meters long. The association panel P2 was divided into four groups based on the maturity group I to IV, and were evaluated for SDS resistance during the summers of 2011 and 2012 in 14 locations including Michigan (Decatur), Iowa (Kanawha and Ames), Minnesota (Waseca and Rosemount), Illinois (Manito, Streator, Fairbury, Beardstown, Urbana, Shawnee town and Valmeyer), Missouri (Sikeston) and Ontario, Canada (Harrow). Four replications per year were grown in a lattice design. SDS was evaluated by scoring disease incidence (DI) and disease severity (DS) at the R6 growth stage, the stage at which pods contain full-size green beans at one of the four uppermost nodes with a completely unrolled leaf [26]. SDS leaf scorch DI was rated from 0% (no disease) to 100% (all plants symptomatic), and DS was measured on a scale from 1 to 9 as described in Additional file 3 (after Bond, J. *unpublished*). The SDS disease index (DX, 0–100) was calculated as DI × DS/9. In panel P1, mean values of DI, DS and DX across replicates and years were used in association analysis throughout the study. The trait distribution for DX and DI was slightly skewed towards susceptible, thus a square root transformation was used to normalize the trait distribution prior to further analysis. The association panel P2 was phenotyped in multiple environments; best linear unbiased predictors (BLUPs) were used for the overall association analysis in panel P2. The BLUPs for each line were calculated with the R package, lme4, using the equation *Y*_*ijk*_ = Line_*k*_ + Environment_*i*_ + Replicate (Environment)_*ij*_ + (Line × Environment)_*ik*_ + ϵ_*ijk*_, where *Y*_*ijk*_ is the observed phenotype for the *k*^th^ line in the *j*^th^ replicate of the *i*^th^ environment; Line_k_ is the random effect of the *k*^th^ line; Environment_*i*_ is the random effect of the *i*^th^ environment; Replicate (Environment)_*ij*_ is the random effect of the *j*^th^ replicate in the *i*^th^ environment; (Environment × Line)_*ik*_ is the random interaction effect of the *i*^th^ environment and the *k*^th^ line, and ϵ_*ijk*_ is the error term. Analysis of variance (ANOVA) for the phenotypic data was performed with the R package, lm(stats) and anova.lm(stats). The heritability estimates were calculated using variance components obtained by ANOVA [27].

### Population genetic analysis

Principal components analysis and neighbor-joining (NJ) trees were applied to infer population stratification. A pairwise distance matrix derived from the Nei’s genetic distance for all polymorphic SNPs was calculated to construct Neighbor-joining trees under PowerMarker version 3.25 [28]. Principal component analysis (PCA) was done using EIGENSTRAT [29] based on 5,578 SNPs and 2,587 SNPs with minor allele frequency (MAF) >20% and physical distance >60 kb for panels P1 and P2, respectively. Kinship matrices were calculated using TASSEL 4.0 [30] to determine relatedness among individuals based on the same sets of SNPs for the two panels [see Additional file 4]. Linkage disequilibrium parameter (*r*^2^) for estimating the degree of LD between pair-wise SNPs (30,345 SNPs for panel P1 and 4,297 SNPs for panel P2 with MAF ≥5%) was calculated using the software TASSEL 4.0 [30] with 1,000 permutations. The LD decay rate was measured as the chromosomal distance at which the average pairwise correlation coefficient (*r*^*2*^) dropped to half its maximum value.

### Genome-wide association analysis

Two different models were used to test associations between the SNPs (MAF >5%) and disease assessment criteria. The first model was a simple model where a general linear model (GLM), containing only the SNP tested as a fixed effect, was used to test the association between the SNP and the disease assessment criteria. The second model is a mixed linear model (MLM) where, in addition to the SNP being tested, PCA matrix and relative kinship matrix were included as fixed and random effects, respectively. The GLM and MLM can be expressed as *y* = *Xα* + *e* and *y* = *Xα* + *Pβ + Kμ + e*, respectively, where *y* is the vector of phenotypic observations, *α is* the vector of SNP effects, *β* is the vector of population structure effects, *μ* is the vector of kinship background effects, *e* is the vector of residual effects, *P* is the PCA matrix relating *y* to *β*, *X* and *K* are incidence matrices of 1 s and 0 s relating *y* to *α* and *μ*, respectively [31]. Top six principal components were used to build up the *P* matrix for population structure correction in the two panels. Analyses were performed by the software TASSEL 4.0 which implemented the EMMA and P3D algorithms to reduce computing time [32]. False discovery rate (FDR) ≤ 0.05 was used to identify significant associations. In order to conduct conditional analyses to test for residual adjacent associations after accounting for a key SNP within the same chromosome, the key SNP was transformed to a numeric value and then added into the MLM as a covariate. A *P*-value threshold of 10^-4^, corresponding to an adjustment for 500 independent tests across the region examined, was used to declare statistical significance at secondary signals.

## Results and discussion

### Genetic diversity and phenotypic variation

Two independent association panels (P1 and P2) were genotyped using Illumina BeadChip containing 52,041 and 5,361 SNPs, respectively. SNPs with MAF of <0.05 and call rate <80% were excluded from further analyses to avoid problems of spurious LD. Final sets of 30,345 and 4,297 high-performing SNPs were used for all analyses. Among these SNPs, samples had an average call rate of >96.5% and between technical replicates yielded >99% pairwise concordances. From these SNPs, we observed an average nucleotide diversity (polymorphism information content or PIC) of 0.281 and 0.284 in panels P1 and P2, respectively. Compared with a previous study [33], these estimates showed that the overall genetic variation of the elite cultivars we studied represents about 80% diversity of soybean landraces. Less than 1.6% of heterozygous genotypes were observed in both panels, which is consistent with the highly inbred nature of cultivated soybean (Table 1). An examination of allele frequency distributions at polymorphic SNPs showed that both panels contained a large number of SNPs with a minor allele frequency (MAF) of <0.1 [see Additional file 5], reflecting the broad genetic diversity in the two association panels.

**Table 1:** **Characteristics of SNPs tested in two association panels**

	Total SNPs	Polymorphic SNPs	MAF ^b^ > 0.05	Density (kb/SNP)	PIC ^c^	Heterozygosity rate
P1 (392^a^)	52,041	39,554	30,345	35	0.281	1.4%
P2 (300)	5,361	5,132	4,297	241	0.284	1.6%

^a^No. of accessions; ^b^MAF, minor allele frequency; ^c^PIC, polymorphism information content.

In both association panels, we observed abundant phenotypic variation in SDS resistance measured by disease incidence (DI), disease severity (DS) and calculated disease index (DX, see Additional file 6). The mean DX distribution ranged from 0 to 96.3 in panel P1 and 0 to 82.0 in panel P2. The broad-sense heritability of DX was higher within two environments (two years in one location) in panel P1 (83%) than that across multiple environments (two years across 14 locations) in panel P2 (average 65%) [see Additional file 6].

### Patterns of linkage disequilibrium

**Figure 1:**
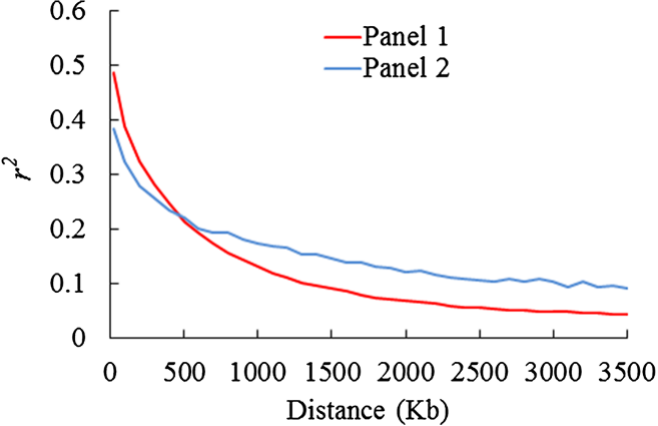
**Genome-wide average LD decay estimated in association panels P1 and P2.** Decay of LD (measured as genotypic *r* ^2^) as a function of distance between SNPs.

To characterize the mapping resolution for genome scans and GWA mapping, we quantified the average extent of genome-wide LD decay distance in panel P1 and P2. These estimates were approximately 270 kb and 460 kb, respectively, where the *r*^*2*^ drops to half its maximum value (0.24 and 0.19, respectively). Given that our average inter-marker distance (density) is 35 kb and 241 kb for the panels P1 and the P2 respectively, we expect to have reasonable power to identify common large effect variants associated with SDS resistance in both association panels. Overall LD decay distance in panel P1 was smaller than that in the panel P2 (Figure 1). LD decay distance in panel P1 was also smaller than previously published values in soybean [33, 34]. This difference may be attributed to smaller sample size and lower genome coverage of markers in P2 and previous studies. Since panel P1 had larger sample size and was genotyped with more SNP markers than panel P2, estimation of LD in panel P1 is more reliable. Linkage disequilibrium decay distance varies over different chromosomes, with 410 kb in chromosome 19, 100–200 kb in chromosomes 3, 4, 9, 10, 11 and 16, and 200–300 kb in the remaining 13 chromosomes in panel P1. These LD decay estimates are slightly higher than that in rice (75-150 kb) [14], but much greater than in maize (1.5-10 kb) [35]. This result is consistent with earlier estimate that LD extends to a much longer distance in self-pollinated species than in cross-pollinated species [12].

### Population structure

**Figure 2:**
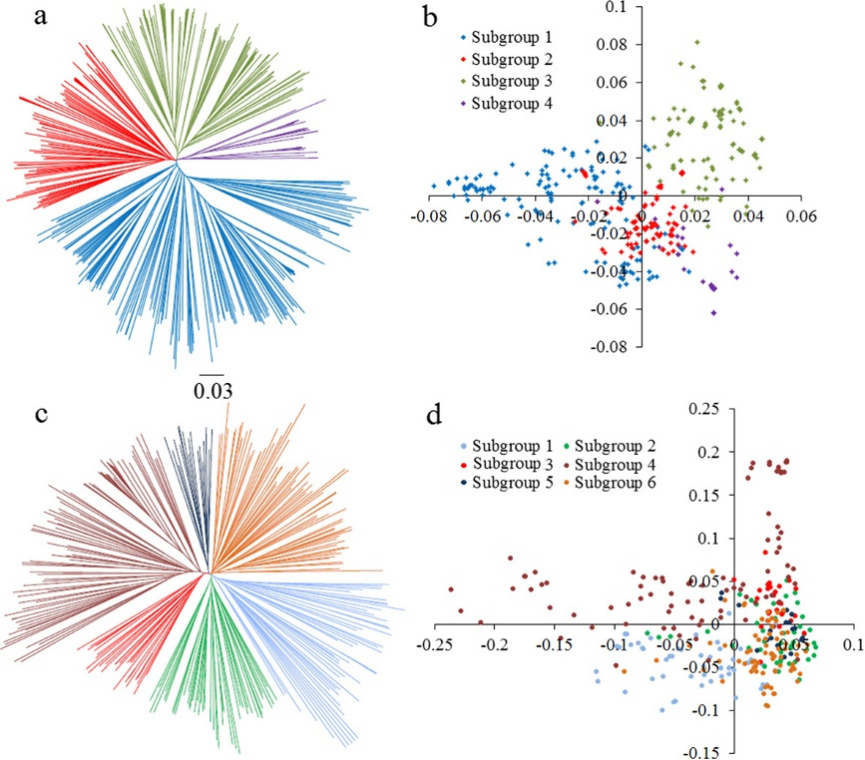
**Population structures of soybean cultivars in association panels P1 and P2.** **(a)** Neighbor-joining tree of 392 accessions in panel P1. The four subgroups identified from the tree are color-coded in **a** and **b**. **(b)** PCA plots of the first two components of 392 accessions in panel P1. **(c)** Neighbor-joining tree of 300 accessions in panel P2. The six subgroups identified from the tree are color-coded in **c** and **d**. **(b)** PCA plots of the first two components of 300 accessions in panel P2.

According to the NJ tree analysis as well as PCA, association panel P1 had 4 genetic subgroups (Figure 2a and b), whereas panel P2 had 6 subgroups (Figure 2c and d). It has previously been suggested that the photoperiod response between different maturity groups may be the primary factor driving differentiation of cultivated soybean [36]. A Chi-square test was used to test whether the SNP-data-based clustering (NJ tree) is associated with maturity-group-based grouping in panel P1 and P2. The results showed very significant association (*P* < 0.0001) between the two grouping factors. Thus the photoperiod response might have driven genetic differentiation among the tested accessions in both panels [see Additional file 7]. The measure of population differentiation, *F*_ST_, was estimated at 0.168 among the four subgroups of panel P1, suggesting a moderate level of differentiation within panel P1 [see Additional file 8]. The population differentiation of 6 subgroups within panel P2 was slightly less (*F*_ST_ = 0.135) but still similar to that between different soybean landraces (*F*_ST_ = 0.130) [33].

### GWA mapping for SDS resistance

**Figure 3:**
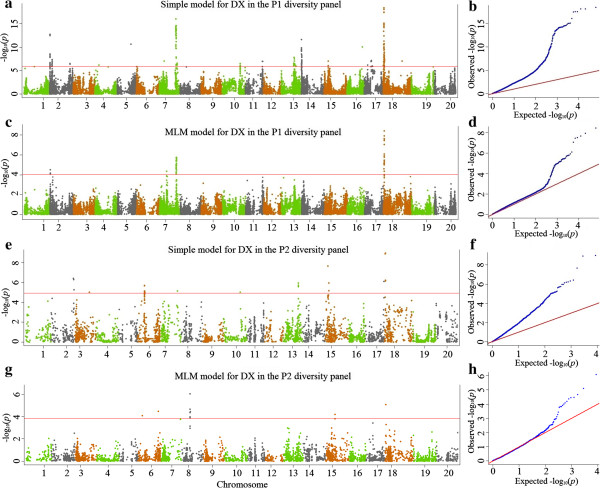
**Genome-wide association study of SDS in the two association panels. (a)** Manhattan plots of the simple model for DX in the association panel P1. The - log10 *P-*values from a genome-wide scan are plotted against the position on each of the 20 chromosomes. The horizontal red line indicates the genome-wide significance threshold (FDR < 0.05). **(b)** Quantile-quantile (QQ) plot of simple model for DX in the association panel P1. **(c)** Manhattan plots of MLM for DX in association panel P1, as in **a**. **(d)** Quantile-quantile plot of MLM for DX in association panel P1. **(e)** Manhattan plots of the simple model for DX in association panel P2, as in **a**. **(f)** Quantile-quantile plot of simple model for DX in the association panel P2. **(g)** Manhattan plots of MLM for DX in association panel P2, as in **a**. **(h)** Quantile-quantile plot of MLM for DX in association panel P2.

Using the GWA strategy to dissect genetic architecture of SDS resistance in the two soybean association panels (P1 and P2), we successfully identified both known associations (candidate genes or QTLs previously reported in soybean), as well as new candidate loci in the soybean genome. The results of significant SNPs discovered in both association panels are summarized in Additional file 9, 10, 11, 12, 13, 14, and Figure 3. As shown in the quantile-quantile (QQ) plots (Figure 3b and f, Additional file 13b and f, and Additional file 14b and f), the distribution of observed -log10 *P*-values from the simple model, which did not include population structure (Q) and familial relatedness (K), departed from the expected distribution under a model of no association with significant inflation of nominal *P*-values. While the MLM method, which includes Q and K, allowed us to reduce the excess low *P*-values for DS, DI and DX (Figure 3d and h, Additional file 13d and h, and Additional file 14d and h). In both association panels, lower inflation of nominal *P*-values was consistently observed when the MLM method was used than when the simple model was used. Therefore, only the results from the analysis with the MLM model are presented below.

**Figure 4:**
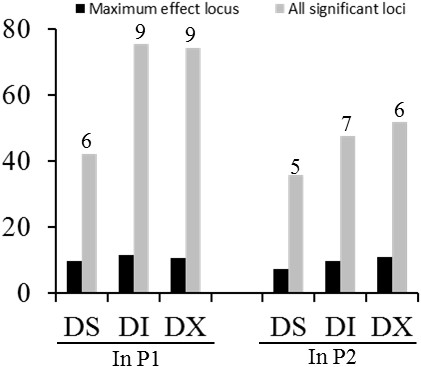
**Contributions of identified loci to phenotypic variance of DS, DI and DX.** Numbers of loci used to estimate contributions to phenotypic variance are indicated at ends of bars.

Of the 52,041 SNPs evaluated in association panel P1, 30, 48 and 56 SNPs were significant, with FDR ≤ 0.05 for DS, DI and DX, respectively (Additional file 9, 10, 11, Figure 3a and c, Additional file 13a and c, and Additional file 14a and c). From the 5,361 SNPs evaluated in association panel P2, 6, 8 and 9 SNPs were significant, with FDR ≤ 0.05 for DS, DI and DX, respectively (Figure 3e and g, Additional file 12, Additional file 13e and g and Additional file 14e and g). To select major QTLs among all the significant SNPs, these SNPs were clumped by using LD block as a criterion [37], and the strongest association within each LD block was kept. After the clumping of SNPs, 20 QTLs for SDS resistance were identified and peak SNPs (strongest associations) are listed in Table 2. The peak SNPs at the identified loci explained approximately 54.5% of the phenotypic variance on average (ranging from 35.7% to 75.4% for different disease assessment criteria, Figure 4). A major QTL on chromosome 18 was found in both association panels (Figure 3).

**Table 2:** **A subset of SNPs significantly associated with SDS resistance and the adjacent candidate genes**

Trait	SNP	Chr ^a^	Position ^b^	*P*	*R* ^2^(%)	QTL ^c^	Candidate genes ^e^
DX(P1)	Gm02-707483	2	707483	3.07 × 10^-5^	5.6	N^d^	PPR repeat (common disease resistance genes, [38])
DI(P2)	ss244884978	2	49773810	3.60 × 10^-4^	6.4	[39]	Cellulose synthase (disease resistance genes, [40])
DX(P2)	ss245842048	6	8979504	8.15 × 10^-5^	7.7	N	Phosphatidylinositol kinase (immune responses, [41])
DX(P2)	ss246038868	6	43945601	3.37 × 10^-5^	5.7	[42]	LRR gene (pathogen recognition, [43])
DX(P1)	Gm07-15654480	7	15654480	4.36 × 10^-5^	5.5	N	Oxysterol binding protein (upregulated in defense response, [44])
DS(P1)	Gm07-36959086	7	36959086	8.86 × 10^-6^	6.5	N	Ubiquitin-like protein (upregulated in defense response, [45])
DX(P2)	ss246580442	8	18469361	8.85 × 10^-7^	10.9	N	Zinc finger (disease resistance genes, [46])
DX(P2)	ss246585278	8	18840490	3.55 × 10^-5^	8.1	N	F-box (defense response, [47])
DI(P1)	Gm09-43648118	9	43648118	6.90 × 10^-5^	11.6	N	Phosphopantetheine (disease response, [48])
DI(P1)	Gm11-37426559	11	37426559	2.23 × 10^-5^	5.6	N	Amino acid transporter (disease resistance genes, [49])
DI(P1)	Gm13-4584015	13	4584015	3.50 × 10^-6^	7.2	[50]	LRR gene (pathogen recognition, [43])
DX(P2)	ss248117124	13	33655223	86 × 10^-4^	5.7	N	Serine/threonine protein kinase (disease defense response, [51])
DI(P1)	Gm14-4636247	14	4636247	6.53 × 10^-5^	5.3	N	Ascorbate oxidase gene (upregulated in defense response, [52])
DI(P2)	ss248566590	15	5978279	7.98 × 10^-4^	5.8	N	Molecular chaperone (plant defense response, [53])
DX(P1)	ss248698930	15	20239752	6.33 × 10^-5^	7.7	N	Serine/threonine protein kinase (disease defense response, [51])
DX(P2)	ss249511029	18	1611921	8.04 × 10^-6^	9.3	[42]	Hypoxia induced protein (disease defense signaling, [54])
DX(P1)	Gm18-1709751	18	1709751	3.79 × 10^-9^	10.6	[42]	Receptor like kinase (disease resistance genes, [11])
DI(P2)	ss249517154	18	2113196	4.04 × 10^-5^	8.3	[42]	unknown
DI(P2)	ss249520656	18	2434513	6.9 × 10^-6^	9.5	[55]	Glycosyltransferase (disease resistance genes, [56])
DI(P1)	Gm19-34890716	19	34890716	2.16 × 10^-5^	5.8	N	Cupins superfamily protein

^a^Chromosome; ^b^Position in base pairs for the peak SNP according to soybean reference sequence of Williams 82; ^c^The candidate gene located in one of the QTL intervals as reported previously and corresponding literature listed in the brackets; ^d^N stands for candidates not located in any known QTL intervals; ^e^A plausible biological candidate gene in the locus or the nearest annotated gene (*Glycine max* Wm82.a1.v1) to the peak SNP.

### QTL confirmation and candidate genes

We compared the positions of the significant SNPs identified in this study with the positions of the QTLs reported in bi-parental mapping studies and found considerable overlap between these SNPs and the reported genes or QTLs for SDS resistance. Of the 20 loci we detected in the two association panels, seven overlapped with previously identified QTLs (Table 2).

**Figure 5:**
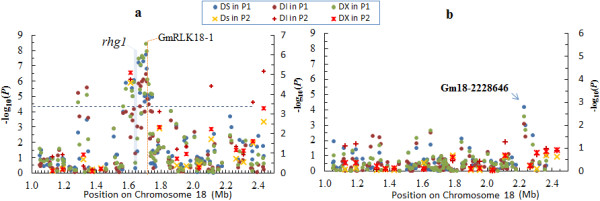
**Regional plots showing association mapping results for SNPs located around** ***Rfs2*** **/** ***Rhg1*** **on chromosome 18.** Before **(a)** and after **(b)** controlling for the effects of peak SNP, negative log10-transformed *P-*values from the MLM are plotted on the left vertical axis for panel P1; Negative log10-transformed *P-*values from the MLM are plotted on the right vertical axis for association panel P2. Blue horizontal dashed lines indicate the genome-wide significance threshold in P1 association panel. Previously identified genes controlling the SDS resistance are labeled.

Notably, one of the overlaps is the QTL *Rfs2/Rhg1* on chromosome 18. This locus consistently contributes more effective coinheritance of resistance to SDS and reduces infestation by SCN [10]. Previous fine map development did not resolve *Rfs2* from *Rhg1*, suggesting that the underlying genes were either very closely linked or pleiotropic [11]. In this study, we did detect a cluster of associations spanning a physical region of 1.2 Mb (1.2-2.4 Mb) around three *Rhg1* genes that were found to contribute to SCN resistance (Figure 5a, Additional file 9, 10, 11, 12) [51]. The cluster of associations also explained a major part of phenotypic variation of SDS resistance in both panels (Additional file 9, 10, 11, 12). If the three *Rhg1* genes were pleiotropic, the peak SNP for SDS resistance should be located either within or in the same LD block with the three *Rhg1* genes. However, the peak SNP (GM18-1709751) was not only located outside of, but also belonged to a different LD block than the three *Rhg1* genes (Additional file 15). One possible explanation for this is that SDS resistance mediated by *Rhg1* is also conferred by copy number variation (CNV) that increases the expression of a set of dissimilar genes. Alternatively, there exist other gene/s mediating SDS resistance that are closely linked with *Rhg1*. In fact, we found that the peak SNP (GM18-1709751) was located at approximately 2.2 kb upstream of *GmRLK18-1*, a gene that encodes a receptor-like kinase, and its resistance allele is sufficient to confer nearly complete resistance to both root and leaf symptoms of SDS [11]. Moreover, there was another significant SNP (GM18-1712832 with *P*-value of 1.2 × 10^-8^ for DX) located within an exon of *GmRLK18-1*. These results support previous studies with regard to the key role of *GmRLK18-1*. However, we cannot exclude the possibility that structural variation in the form of CNV may have functional importance and thus contribute to SDS resistance that is not captured by our SNPs.

We searched for additional independently associated SNP variants near the *Rfs2/Rhg1* locus by conditioning on the peak SNP (GM18-1709751) at the *Rfs2/Rhg1* loci. At 519 kb downstream of *Rfs2/Rhg1*, we found an independently associated SNP variant, Gm18-2228646 with *P*-value of 6.5 × 10^-5^ in the conditional model, as measured by DS. This SNP is located at 44 bp downstream of a gene encoding an aquaporin transporter (Figure 5b). Therefore, our results provide evidence for the presence of a regulating gene other than *GmRLK18-*1 that is associated with SDS resistance on chromosome 18.

Besides the *Rfs2/Rhg1* region, we refined the mapping location with other significant SNPs within or adjacent to previously reported QTLs (Table 2). Notably, we repeatedly detected a cluster of associations, measured by DS, DI and DX, spanning a physical region of 0.7 Mb (36.5 to 37.2 Mb) near the *Rzd* locus (resistance to zygote death) on chromosome 7 in panel P1. The *Rzd* locus contributed to SCN resistance and was strictly co-inherited in phase with *Rfs2/Rhg1* in an earlier study [57]. However, not all of the QTLs detected in previous bi-parental populations were detected in our association panels. The reason for failure to detect them may be that root infection severity is not included in our disease assessment criteria, so those QTLs associated with resistance to root infection cannot be identified. Alternatively, some QTLs may segregate at low frequency or not at all in our association panels, or the SNP coverage in this study is still insufficient to capture all of the haplotypes present in the diverse soybean varieties. On the other hand, we found 13 novel QTLs. Compared with the 7 loci within intervals of known QTLs, the 13 new loci are slightly weaker in terms of average P-value (6.47 × 10^-5^ vs 7.54 × 10^-4^) and explained phenotypic variance (8.14% vs 7.06%). However, some of them explain as much as, or even more phenotypic variance than that of known QTLs (Table 2). We checked whether these new QTLs were near loci for determinacy, maturity date, leaf, stem or root morphology and found that one new QTL at 33.6 Mb on chromosome 13 was within the interval containing QTLs for plant height and stem strength. Another new QTL at 34.8 Mb on chromosome 19 was located approximately 2 Mb downstream of a locus related with flowering time and leaf morphology. No new QTLs were found near loci related to determinacy, maturity date, or root morphology. To further validate these new loci, we developed five recombinant inbred line (RIL) populations and are currently conducting a confirmation study. To date, we have conducted conventional QTL mapping in three of the five RIL populations. Five out of the 13 novel QTLs have been validated in the three RIL populations (data not shown). Undoubtedly, the major loci identified in this study can be used to improve soybean for SDS resistance.

When we checked candidate genes containing or immediately adjacent to the significant SNPs, we found that diverse types of genes are probably involved in natural variation for soybean SDS resistance (Table 2). For instance, we identified one pentatricopeptide repeat (PPR) gene, which has certain features in common with disease resistance genes (R genes) [38]. We also identified two genes encoding leucine-rich repeat (LRR) domains, which are important in plant responses to a variety of external stimuli including pathogens (Table 2 and ref. 44). A gene with similarity to ubiquitin-like protein, which is required for host and nonhost disease resistance in plants [45], was also identified. Several other SNPs were within or adjacent to sequences annotated as homologous to genes previously shown to be involved in plant disease resistance (Table 2). Follow-up studies will focus on validating effects of these genes, uncovering the molecular mechanisms of complex SDS resistance in soybean and integrating this knowledge to dissect mechanisms underlying quantitative resistance to soil-borne pathogens.

## Conclusions

In this study, GWA mapping with correction for population structure and cryptic relatedness identified multiple novel loci and refined the map locations of known loci related to SDS resistance in soybean. This information not only demonstrates that GWA mapping can be used as a powerful tool for dissecting disease resistance mechanisms in soybean, but also provides valuable markers for developing soybean cultivars with durable resistance against SDS. Moreover, the candidate genes containing these SNP loci represent promising targets for further efforts to pinpoint causal variants and to clarify how the implicated genes affect SDS resistance in soybean.

### Availability of supporting data

The data sets supporting the results of this article are included within this article and its additional files.

## Electronic supplementary material

Additional file 1: **Soybean germplasm accessions analyzed in this study.** Information is given in this file for each accession, including accession name, origin, maturity group and subpopulation ancestry based Neighbor-joining trees. (XLSX 34 KB)

Additional file 2: **SNPs Distribution of each chromosome on SoySNP 50 k (a) and SoySNP 6 k (b)BeadChip used in genotyping for panel P1 and P2, respectively.** This figure is a color index showing the SNP distribution and density of 20 chromosomes on SoySNP 50 k (a) and SoySNP 6 k (b) BeadChip. (DOCX 79 KB)

Additional file 3: **Scale used for phenotyping sudden death syndrome disease severity (DS).** Disease incidence (DI) is the percentage of plants in the plot showing leaf symptoms. Disease index (DX) = (DI × DS)/9. (DOCX 156 KB)

Additional file 4: **Kinship value between individual accessions among panels P1 (a) and P2 (b).** Individuals are ordered according to their order listed in Additional file 1. Pairwise kinship values are shown as color-index heat map. (DOCX 2 MB)

Additional file 5: **The distributions of minor allele frequencies in P1 (a) and P2 (b) association panels.** Two histograms, a for panel P1 and b for panel P2, showing the distributions of minor allele frequencies in two association panels. (DOCX 92 KB)

Additional file 6: **Phenotypic variation, heritability and correlation analysis in the two association panels.** Descriptive statistics information, including mean, range, standard deviation, source of variation and correlation coefficient for DS, DI and DX. (DOCX 17 KB)

Additional file 7: **Distribution of accessions in each subgroup based on genetic distance in panel P1 and P2.** Two-way classification of all accessions, with SNP-data-based clustering (NJ tree) at the top and the maturity-group-based grouping clusters at the left. (DOCX 15 KB)

Additional file 8: **The population differentiation statistics (** ***F***_**ST**_ **) among subpopulation in panel P1 and P2.** Pairwise population differentiation index (*F*st) as well as corresponding significant levels are list in this table. (DOCX 16 KB)

Additional file 9: **Associations (FDR < 0.05) identified by GWA mapping for DS in association panel P1.** Information of significantly associated SNPs, including name, physical position and phenotypic variation explained by the SNP, is reported in this table. (DOCX 17 KB)

Additional file 10: **Associations (FDR < 0.05) identified by GWAS for DI in association panel P1.** Information of significantly associated SNPs, including name, physical position and phenotypic variation explained by the SNP, is reported in this table. (DOCX 19 KB)

Additional file 11: **Associations (FDR < 0.05) identified by GWAS for DX in association panel P1.** Information of significantly associated SNPs, including name, physical position and phenotypic variation explained by the SNP, is reported in this table. (DOCX 20 KB)

Additional file 12: **Associations (FDR < 0.05) identified by GWAS in association panel P2.** Information of significantly associated SNPs, including name, physical position and phenotypic variation explained by the SNP, are reported in this table. (DOCX 18 KB)

Additional file 13: **Genome-wide association study of DS in the two association panels.** (a) Manhattan plots of the simple model for DS in association panel P1. The - log10 *P* values from a genome-wide scan are plotted against the position on each of the 20 chromosomes. The horizontal red line indicates the genome-wide significance threshold (FDR < 0.05). (b) Quantile-quantile plot of simple model for DS in the association panel P1. (c) Manhattan plots of MLM for DX in association panel P2, as in a. (d) Quantile-quantile plot of MLM for DS in the panel P1. (e) Manhattan plots of the simple model for DS in association panel P2, as in a. (f) Quantile-quantile plot of simple model for DS in the panel P2. (g) Manhattan plots of MLM for DS in the panel P1, as in a. (h) Quantile-quantile plot of MLM for DS in association panel P2. (DOCX 965 KB)

Additional file 14: **Genome-wide association study of DI in the two association panels.** (a) Manhattan plots of the simple model for DI in association panel P1. The - log10 *P* values from a genome-wide scan are plotted against the position on each of the 20 chromosomes. The horizontal red line indicates the genome-wide significance threshold (FDR < 0.05). (b) Quantile-quantile plot of simple model for DI in the association panel P1. (c) Manhattan plots of MLM for DX in association panel P1, as in a. (d) Quantile-quantile plot of MLM for DI in association panel P1. (e) Manhattan plots of the simple model for DI in association panel P2, as in a. (f) Quantile-quantile plot of simple model for DI in the association panel P2. (g) Manhattan plots of MLM for DI in association panel P2, as in a. (h) Quantile-quantile plot of MLM for DI in association panel P2. (DOCX 1 MB)

Additional file 15: **Regional plots showing association mapping results for SNPs located around** ***Rfs2*** **/** ***Rhg1*** **on chromosome 18.** Negative log10-transformed *P-*values from the MLM are plotted on the left vertical axis for association panel P1; Negative log10-transformed *P-*values from the MLM are plotted on the right vertical axis for association panel P2. Blue horizontal dashed lines indicate the genome-wide significance threshold in association panel P1. Previously identified genes controlling the traits are labeled. (DOCX 94 KB)

## Abbreviations

SDS: Sudden death syndrome
SCN: Soybean cyst nematode
DI: Disease incidence
DS: Disease severity
DX: Disease index
GWA: Genome-wide association
SNPs: Single nucleotide polymorphisms
LD: Linkage disequilibrium
QTL: Quantitative trait locus
SSR: Simple sequence repeat
GLM: General linear model
MLM: Mixed linear model.
