# Supplementary material for: Genome-wide association mapping of quantitative resistance to sudden death syndrome in soybean
Source: BMC Genomics. 2014 Sep 23;15(1):809. doi: 10.1186/1471-2164-15-809 (PMC4189206; doi:10.1186/1471-2164-15-809)
Supplement: Supplementary file 2 — Additional file 2: SNPs Distribution of each chromosome on SoySNP 50 k (a) and SoySNP 6 k (b)BeadChip used in genotyping for panel P1 and P2, respectively. This figure is a color index showing the SNP distribution and density of 20 chromosomes on SoySNP 50 k (a) and SoySNP 6 k (b) BeadChip. (DOCX 79 KB) [file 12864_2014_6491_MOESM2_ESM.docx]

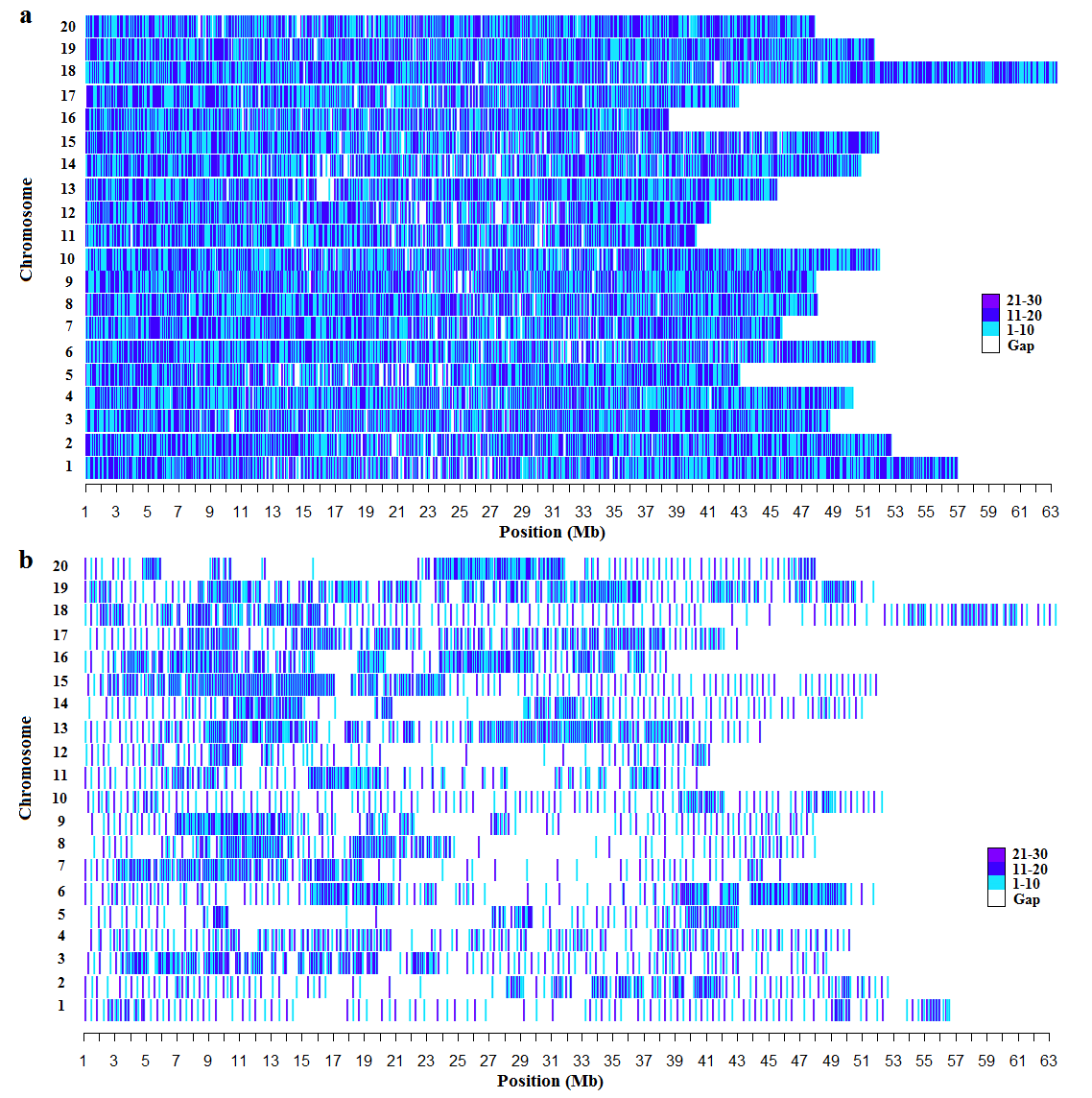


**Additional file 2**. SNPs Distribution of each chromosome on SoySNP 50k (a) and SoySNP 6k(b)-chip used in genotyping for panel P1 and P2 respectively. The number of SNP per 10 kb is shown as color index.
