# Supplementary material for: Genome-wide association mapping of quantitative resistance to sudden death syndrome in soybean
Source: BMC Genomics. 2014 Sep 23;15(1):809. doi: 10.1186/1471-2164-15-809 (PMC4189206; doi:10.1186/1471-2164-15-809)
Supplement: Supplementary file 3 — Additional file 3: Scale used for phenotyping sudden death syndrome disease severity (DS). Disease incidence (DI) is the percentage of plants in the plot showing leaf symptoms. Disease index (DX) = (DI × DS)/9. (DOCX 156 KB) [file 12864_2014_6491_MOESM3_ESM.docx]

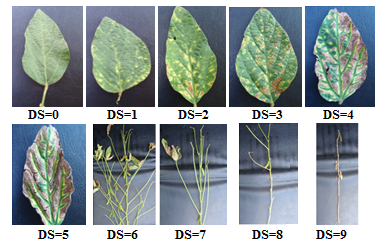


| DS=0: No evidence of sudden death syndrome |
| --- |
| DS=1: 1%-10% of the leaf surface chlorotic or 1%-5% necrotic |
| DS=2: 11%-20% of the leaf surface chlorotic or 6%-10% necrotic |
| DS=3: 21%-40% of the leaf surface chlorotic or 11%-20% necrotic |
| DS=4: 41%-60% of the leaf surface chlorotic or 21%-40% necrotic |
| DS=5: greater than 60% chlorotic or greater than 40% necrotic |
| DS=6: up to 1/3 premature defoliation |
| DS=7: 1/3 to 2/3 premature defoliation |
| DS=8: greater than 2/3 premature defoliation |
| DS=9: plant death before normal defoliation due to senescence |

**Additional file 3**. Scale used for phenotyping sudden death syndrome disease severity (DS) (after Bond, J. unpublished). Disease incidence (DI) is the percentage of plants in the plot showing leaf symptoms. Disease index (DX) = (DI×DS)/9
