# Supplementary material for: Genome-wide association mapping of quantitative resistance to sudden death syndrome in soybean
Source: BMC Genomics. 2014 Sep 23;15(1):809. doi: 10.1186/1471-2164-15-809 (PMC4189206; doi:10.1186/1471-2164-15-809)
Supplement: Supplementary file 4 — Additional file 4: Kinship value between individual accessions among panels P1 (a) and P2 (b). Individuals are ordered according to their order listed in Additional file 1. Pairwise kinship values are shown as color-index heat map. (DOCX 2 MB) [file 12864_2014_6491_MOESM4_ESM.docx]

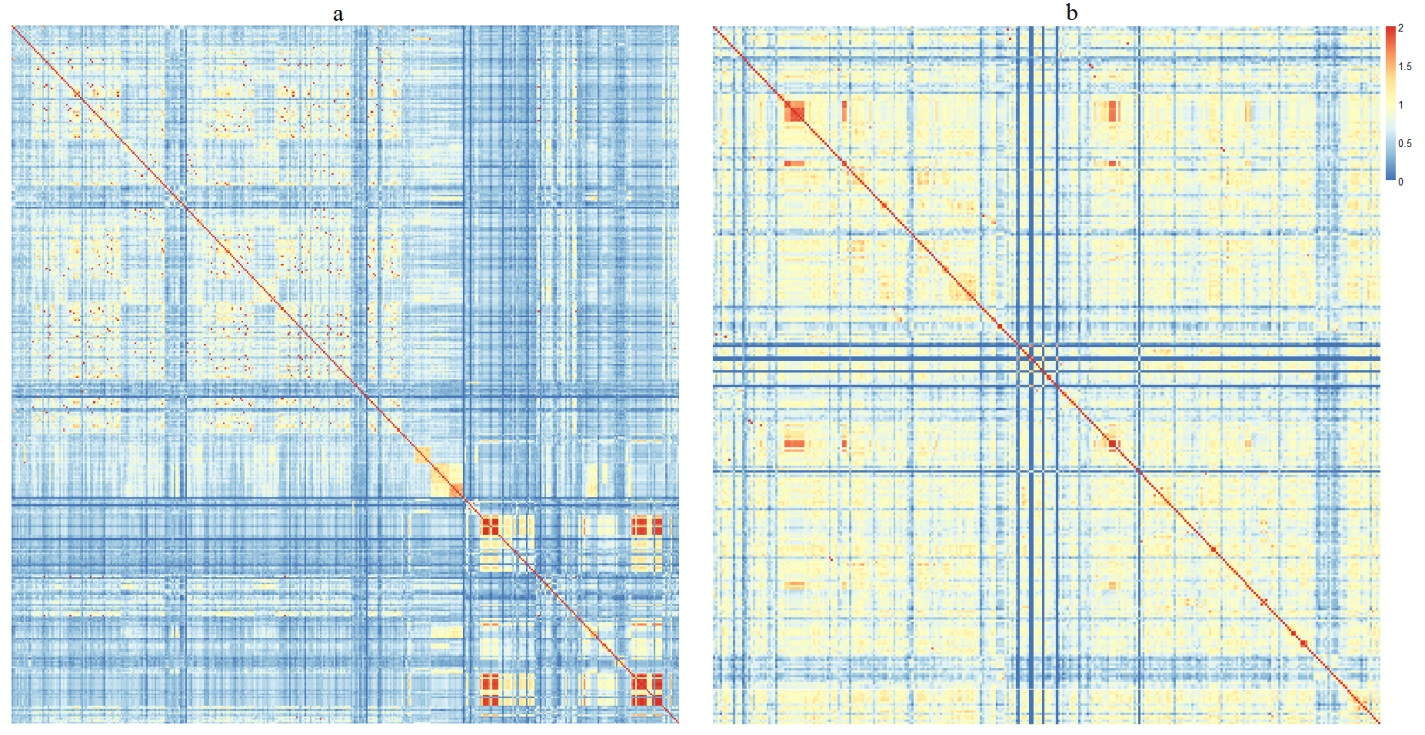

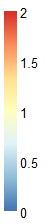


**Additional file 4**. Kinship value between individual accessions among P1(a) and P2(b). Individuals are ordered according their order listed in Additional file1. Each pixel in the square indicates the kinship value of corresponding individual pair as shown in the right side coloured bar.
