# Supplementary figures and images for: Genome-wide association mapping of quantitative resistance to sudden death syndrome in soybean
Source: BMC Genomics. 2014 Sep 23;15(1):809. doi: 10.1186/1471-2164-15-809 (PMC4189206; doi:10.1186/1471-2164-15-809)

**
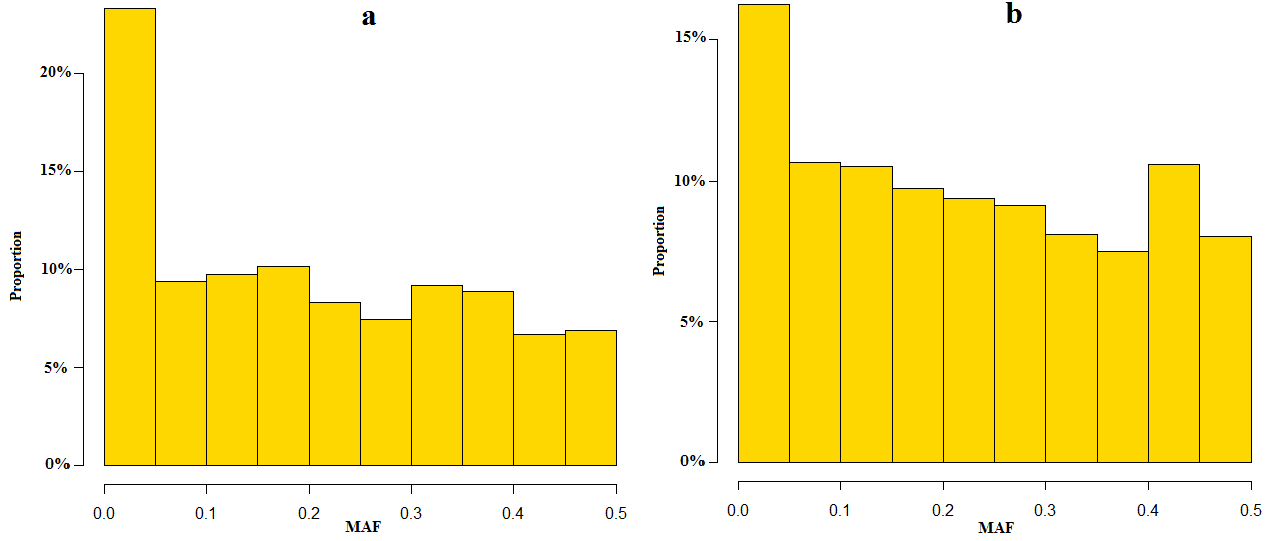
**

**Additional file 5**. The distributions of minor allele frequencies in P1(a) and P2(b) association panel.

Supplement: Supplementary file 5 — Additional file 5: The distributions of minor allele frequencies in P1 (a) and P2 (b) association panels. Two histograms, a for panel P1 and b for panel P2, showing the distributions of minor allele frequencies in two association panels. (DOCX 92 KB) [file 12864_2014_6491_MOESM5_ESM.docx]
