# Supplementary material for: Genome-wide association mapping of quantitative resistance to sudden death syndrome in soybean
Source: BMC Genomics. 2014 Sep 23;15(1):809. doi: 10.1186/1471-2164-15-809 (PMC4189206; doi:10.1186/1471-2164-15-809)
Supplement: Supplementary file 6 — Additional file 6: Phenotypic variation, heritability and correlation analysis in the two association panels. Descriptive statistics information, including mean, range, standard deviation, source of variation and correlation coefficient for DS, DI and DX. (DOCX 17 KB) [file 12864_2014_6491_MOESM6_ESM.docx]

**Additional file 6. Phenotypic variation, heritability and correlation analysis in the two association panels**

| Category | Source of variation | P1 association panel | | |  | | P2 association panel | | |
| --- | --- | --- | --- | --- | --- | --- | --- | --- | --- |
|  |  | DS | DI | DX |  | DS | | DI | DX |
| Descriptive  statistics | Range | 0.0–8.7 | 0.0–100.0 | 0.0–96.3 |  | 0.0-7.6 | | 0.0-100.0 | 0.0-82.2 |
|  | Mean ± s.d.* | 4.2 ± 1.7 | 71.2±27.9 | 38.5±23.5 |  | 2.2±1.3 | | 37.7±28.4 | 13.3±13.2 |
| ANOVA | G^†^ | ** | ** | ** |  | ** | | ** | ** |
|  | G×E^‡^ | ns.^§^ | ns. | ns. |  | ** | | ** | ** |
|  | Heritability | 0.82 | 0.80 | 0.83 |  | 0.58 | | 0.67 | 0.65 |
| Correlation  coefficient ^c^ | DS | – | 0.76** | 0.96** |  | – | | 0.61** | 0.85** |
|  | DI |  | – | 0.86** |  |  | | – | 0.84** |
|  | DX |  |  | – |  |  | |  | – |

*s.d., standard deviation; ^†^G, Genotype across different environments; ^‡^ G×E , Genotype ×environment(for P1, including genotype× year; for P2, including genotype× year , genotype× location, genotype × year × location);; ^§^ns, not significant;**Significant at *P* < 0.01
