# Supplementary material for: Genome-wide association mapping of quantitative resistance to sudden death syndrome in soybean
Source: BMC Genomics. 2014 Sep 23;15(1):809. doi: 10.1186/1471-2164-15-809 (PMC4189206; doi:10.1186/1471-2164-15-809)
Supplement: Supplementary file 7 — Additional file 7: Distribution of accessions in each subgroup based on genetic distance in panel P1 and P2. Two-way classification of all accessions, with SNP-data-based clustering (NJ tree) at the top and the maturity-group-based grouping clusters at the left. (DOCX 15 KB) [file 12864_2014_6491_MOESM7_ESM.docx]

**Additional file 7. Distribution of accessions in each subgroup based on genetic distance in panel P1 and P2**

|  | Subpopulation of P1 | | | |  | Subpopulation of P2 | | | | | |
| --- | --- | --- | --- | --- | --- | --- | --- | --- | --- | --- | --- |
|  | 1 | 2 | 3 | 4 |  | 1 | 2 | 3 | 4 | 5 | 6 |
| MG* I | 52 | 5 | 2 | 0 |  | 29 | 3 | 6 | 2 | 4 | 9 |
| MG II | 94 | 41 | 50 | 10 |  | 10 | 14 | 11 | 25 | 12 | 23 |
| MG III | 24 | 39 | 59 | 16 |  | 9 | 14 | 2 | 22 | 2 | 23 |
| MG IV | - | - | - | - |  | 2 | 17 | 8 | 35 | 0 | 18 |
| *χ*^2^= 90.63,*χ*^2^_0.01,6_ = 16.81, *P*<0.001 | | | | | | χ^2^= 103.01,*χ*^2^_0.01,15_ = 30.58 , *P*<0.001 | | | | | |

*MG, maturity group.
