# Supplementary material for: Genome-wide association mapping of quantitative resistance to sudden death syndrome in soybean
Source: BMC Genomics. 2014 Sep 23;15(1):809. doi: 10.1186/1471-2164-15-809 (PMC4189206; doi:10.1186/1471-2164-15-809)
Supplement: Supplementary file 8 — Additional file 8: The population differentiation statistics ( F ST ) among subpopulation in panel P1 and P2. Pairwise population differentiation index (Fst) as well as corresponding significant levels are list in this table. (DOCX 16 KB) [file 12864_2014_6491_MOESM8_ESM.docx]

**Additional file 8.The population-differentiation statistics (*F*_ST_) among subpopulation in panel P1 and P2.**

|  |  | Subpopulation of P1 | | |  |  |  | Subpopulation of P2 | | | | | |  |
| --- | --- | --- | --- | --- | --- | --- | --- | --- | --- | --- | --- | --- | --- | --- |
|  |  | 1 | 2 | 3 | 4 |  |  | 1 | 2 | 3 | 4 | 5 | 6 |  |
|  | 1 | 0 |  |  |  |  | 1 | 0 |  |  |  |  |  |  |
|  | 2 | 0.104** | 0 |  |  |  | 2 | 0.137** | 0 |  |  |  |  |  |
|  | 3 | 0.180** | 0.157** | 0 |  |  | 3 | 0.161** | 0.147** | 0 |  |  |  |  |
|  | 4 | 0.190** | 0.154** | 0.224** | 0 |  | 4 | 0.118** | 0.144** | 0.106 | 0 |  |  |  |
|  |  |  |  |  |  |  | 5 | 0.164 | 0.179** | 0.147** | 0.174 | 0 |  |  |
|  |  |  |  |  |  |  | 6 | 0.107** | 0.099** | 0.099** | 0.122** | 0.126 | 0 |  |

. **, *P* < 0.01
