# Supplementary material for: Genome-wide association mapping of quantitative resistance to sudden death syndrome in soybean
Source: BMC Genomics. 2014 Sep 23;15(1):809. doi: 10.1186/1471-2164-15-809 (PMC4189206; doi:10.1186/1471-2164-15-809)
Supplement: Supplementary file 11 — Additional file 11: Associations (FDR < 0.05) identified by GWAS for DX in association panel P1. Information of significantly associated SNPs, including name, physical position and phenotypic variation explained by the SNP, is reported in this table. (DOCX 20 KB) [file 12864_2014_6491_MOESM11_ESM.docx]

**Additional file 11. Associations (FDR<0.05) identified by GWAS for DX in association panel P1**

| Site Name | Chromosome | Position | Allele | MAF | *P* value | *R*^2^ |
| --- | --- | --- | --- | --- | --- | --- |
| Gm02-706966 | 2 | 706966 | A/G | 0.42746 | 7.65E-05 | 0.05002 |
| Gm02-707483 | 2 | 707483 | G/A | 0.41948 | 3.07E-05 | 0.0555 |
| Gm02-708417 | 2 | 708417 | T/G | 0.40909 | 6.86E-05 | 0.05116 |
| Gm07-15654480 | 7 | 15654480 | A/G | 0.32846 | 4.36E-05 | 0.0549 |
| Gm07-36480188 | 7 | 36480188 | G/A | 0.38961 | 1.22E-05 | 0.06131 |
| Gm07-36483418 | 7 | 36483418 | G/A | 0.39203 | 1.17E-05 | 0.05998 |
| Gm07-36488859 | 7 | 36488859 | A/C | 0.39203 | 1.17E-05 | 0.05998 |
| Gm07-36498461 | 7 | 36498461 | C/T | 0.39119 | 1.10E-05 | 0.06125 |
| Gm07-36511460 | 7 | 36511460 | G/T | 0.39276 | 8.63E-06 | 0.06173 |
| Gm07-36526356 | 7 | 36526356 | G/A | 0.19764 | 1.86E-06 | 0.07286 |
| Gm07-36534897 | 7 | 36534897 | T/G | 0.39221 | 1.34E-05 | 0.05966 |
| Gm07-36539495 | 7 | 36539495 | C/T | 0.39304 | 1.25E-05 | 0.05979 |
| Gm07-36540823 | 7 | 36540823 | C/A | 0.39433 | 4.97E-05 | 0.05265 |
| Gm07-36560926 | 7 | 36560926 | T/C | 0.3946 | 1.73E-05 | 0.05786 |
| Gm07-36605671 | 7 | 36605671 | C/T | 0.3946 | 1.10E-05 | 0.06053 |
| Gm07-36615942 | 7 | 36615942 | T/C | 0.38731 | 8.02E-05 | 0.05033 |
| Gm07-36649899 | 7 | 36649899 | T/C | 0.39556 | 8.28E-05 | 0.05026 |
| Gm07-36669741 | 7 | 36669741 | G/A | 0.38351 | 5.78E-05 | 0.05302 |
| Gm07-36671857 | 7 | 36671857 | C/T | 0.38624 | 1.85E-05 | 0.0603 |
| Gm07-36706803 | 7 | 36706803 | T/C | 0.38095 | 1.29E-05 | 0.06217 |
| Gm07-36767621 | 7 | 36767621 | A/G | 0.39046 | 2.73E-05 | 0.05572 |
| Gm07-36782624 | 7 | 36782624 | T/G | 0.39018 | 1.79E-05 | 0.05817 |
| Gm07-36787244 | 7 | 36787244 | G/A | 0.38831 | 2.32E-05 | 0.05733 |
| Gm07-36814162 | 7 | 36814162 | G/A | 0.38672 | 3.76E-06 | 0.06746 |
| Gm07-36838943 | 7 | 36838943 | T/C | 0.38451 | 9.85E-06 | 0.06268 |
| Gm07-36843590 | 7 | 36843590 | C/T | 0.37922 | 1.78E-05 | 0.05911 |
| Gm07-36848705 | 7 | 36848705 | A/G | 0.39323 | 4.29E-06 | 0.06639 |
| Gm07-36959086 | 7 | 36959086 | T/C | 0.38976 | 1.75E-06 | 0.07241 |
| Gm07-36972752 | 7 | 36972752 | T/C | 0.39018 | 3.73E-06 | 0.06665 |
| Gm07-36997691 | 7 | 36997691 | T/C | 0.39637 | 2.16E-06 | 0.0708 |
| Gm07-36998381 | 7 | 36998381 | C/T | 0.37918 | 3.03E-06 | 0.06743 |
| Gm07-37010895 | 7 | 37010895 | G/T | 0.39018 | 1.06E-05 | 0.06133 |
| Gm07-37096617 | 7 | 37096617 | A/G | 0.3899 | 7.03E-06 | 0.06308 |
| Gm07-37120820 | 7 | 37120820 | G/T | 0.37958 | 3.28E-05 | 0.05663 |
| Gm07-37129343 | 7 | 37129343 | G/A | 0.375 | 4.21E-06 | 0.06966 |
| Gm18-1286527 | 18 | 1286527 | A/G | 0.40181 | 2.53E-05 | 0.05585 |
| Gm18-1340190 | 18 | 1340190 | T/C | 0.4699 | 7.91E-06 | 0.06274 |
| Gm18-1567581 | 18 | 1567581 | G/A | 0.10392 | 4.47E-05 | 0.07613 |
| Gm18-1582570 | 18 | 1582570 | C/T | 0.47105 | 3.39E-06 | 0.06773 |
| Gm18-1620585 | 18 | 1620585 | C/T | 0.4752 | 4.69E-06 | 0.06576 |
| Gm18-1625693 | 18 | 1625693 | A/G | 0.48481 | 1.01E-05 | 0.06672 |
| Gm18-1634453 | 18 | 1634453 | G/A | 0.4973 | 1.24E-06 | 0.07936 |
| Gm18-1663298 | 18 | 1663298 | A/G | 0.48052 | 3.51E-08 | 0.08253 |
| Gm18-1671483 | 18 | 1671483 | A/G | 0.43081 | 3.73E-06 | 0.06721 |
| Gm18-1674972 | 18 | 1674972 | C/T | 0.45778 | 8.73E-08 | 0.08982 |
| Gm18-1682082 | 18 | 1682082 | A/G | 0.45822 | 2.26E-08 | 0.09604 |
| Gm18-1690566 | 18 | 1690566 | A/G | 0.46306 | 3.12E-08 | 0.09392 |
| Gm18-1699011 | 18 | 1699011 | T/C | 0.43947 | 6.52E-06 | 0.06422 |
| Gm18-1701594 | 18 | 1701594 | C/T | 0.43081 | 6.89E-06 | 0.06387 |
| Gm18-1709751 | 18 | 1709751 | G/A | 0.48052 | 3.79E-09 | 0.10607 |
| Gm18-1712832 | 18 | 1712832 | T/C | 0.48037 | 1.20E-08 | 0.09947 |
| Gm18-1718002 | 18 | 1718002 | G/A | 0.45312 | 8.18E-07 | 0.07609 |
| Gm18-1726316 | 18 | 1726316 | T/G | 0.45419 | 1.62E-06 | 0.07157 |
| Gm18-1737465 | 18 | 1737465 | T/C | 0.43099 | 6.90E-06 | 0.06374 |
| Gm18-1745477 | 18 | 1745477 | T/C | 0.42857 | 7.38E-06 | 0.06342 |
| Gm18-1861988 | 18 | 1861988 | T/C | 0.34704 | 9.42E-05 | 0.04881 |
