# Supplementary material for: Genome-wide association mapping of quantitative resistance to sudden death syndrome in soybean
Source: BMC Genomics. 2014 Sep 23;15(1):809. doi: 10.1186/1471-2164-15-809 (PMC4189206; doi:10.1186/1471-2164-15-809)
Supplement: Supplementary file 12 — Additional file 12: Associations (FDR < 0.05) identified by GWAS in association panel P2. Information of significantly associated SNPs, including name, physical position and phenotypic variation explained by the SNP, are reported in this table. (DOCX 18 KB) [file 12864_2014_6491_MOESM12_ESM.docx]

**Additional file 12. Associations (FDR<0.05) identified by GWAS in association panel P2**

| Disease assessment criteria | SNP Name | Chromosome | Position | Allele | MAF | *P* value | *R*^2^ |
| --- | --- | --- | --- | --- | --- | --- | --- |
| DS | ss245842048 | 6 | 8979504 | T/C | 0.494881 | 1.67E-04 | 0.061 |
|  | ss246580442 | 8 | 18469361 | T/C | 0.094178 | 5.69E-05 | 0.068 |
|  | ss246584117 | 8 | 18732374 | T/C | 0.160345 | 2.70E-04 | 0.059 |
|  | ss248696122 | 15 | 20077360 | T/G | 0.072881 | 1.50E-04 | 0.060 |
|  | ss248697143 | 15 | 20150463 | T/C | 0.077181 | 1.50E-04 | 0.060 |
|  | ss249511029 | 18 | 1611921 | T/C | 0.397569 | 2.79E-05 | 0.074 |
| DI | ss244884978 | 2 | 49773810 | A/C | 0.186007 | 3.60E-04 | 0.064 |
|  | ss248117124 | 13 | 33655223 | A/C | 0.0625 | 8.61E-04 | 0.057 |
|  | ss248566590 | 15 | 5978279 | A/G | 0.303819 | 7.98E-04 | 0.058 |
|  | ss249511029 | 18 | 1611921 | T/C | 0.397569 | 1.82E-05 | 0.087 |
|  | ss249513185 | 18 | 1790730 | A/G | 0.256944 | 8.15E-04 | 0.058 |
|  | ss249517154 | 18 | 2113196 | A/C | 0.487676 | 4.04E-05 | 0.083 |
|  | ss249519978 | 18 | 2368270 | A/G | 0.42931 | 2.72E-04 | 0.067 |
|  | ss249520656 | 18 | 2434513 | A/G | 0.439446 | 6.87E-06 | 0.095 |
| DX | ss245842048 | 6 | 8979504 | T/C | 0.494881 | 8.15E-05 | 0.077 |
|  | ss246052224 | 6 | 43945601 | A/G | 0.128378 | 3.37E-05 | 0.082 |
|  | ss246578369 | 8 | 18324561 | T/C | 0.441729 | 0.0001 | 0.077 |
|  | ss246580442 | 8 | 18469361 | T/C | 0.094178 | 8.8E-07 | 0.109 |
|  | ss246584117 | 8 | 18732374 | T/C | 0.160345 | 2.18E-05 | 0.087 |
|  | ss246584798 | 8 | 18783086 | A/G | 0.166102 | 4.36E-05 | 0.080 |
|  | ss246585278 | 8 | 18840490 | A/G | 0.161565 | 3.55E-05 | 0.082 |
|  | ss248698930 | 15 | 20239752 | T/C | 0.070946 | 6.34E-05 | 0.077 |
|  | ss249511029 | 18 | 1611921 | T/C | 0.397569 | 8.05E-06 | 0.093 |
