# Supplementary material for: Genome-wide association mapping of quantitative resistance to sudden death syndrome in soybean
Source: BMC Genomics. 2014 Sep 23;15(1):809. doi: 10.1186/1471-2164-15-809 (PMC4189206; doi:10.1186/1471-2164-15-809)
Supplement: Supplementary file 13 — Additional file 13: Genome-wide association study of DS in the two association panels. (a) Manhattan plots of the simple model for DS in association panel P1. The - log10 P values from a genome-wide scan are plotted against the position on each of the 20 chromosomes. The horizontal red line indicates the genome-wide significance threshold (FDR < 0.05). (b) Quantile-quantile plot of simple model for DS in the association panel P1. (c) Manhattan plots of MLM for DX in association panel P2, as in a. (d) Quantile-quantile plot of MLM for DS in the panel P1. (e) Manhattan plots of the simple model for DS in association panel P2, as in a. (f) Quantile-quantile plot of simple model for DS in the panel P2. (g) Manhattan plots of MLM for DS in the panel P1, as in a. (h) Quantile-quantile plot of MLM for DS in association panel P2. (DOCX 965 KB) [file 12864_2014_6491_MOESM13_ESM.docx]

**
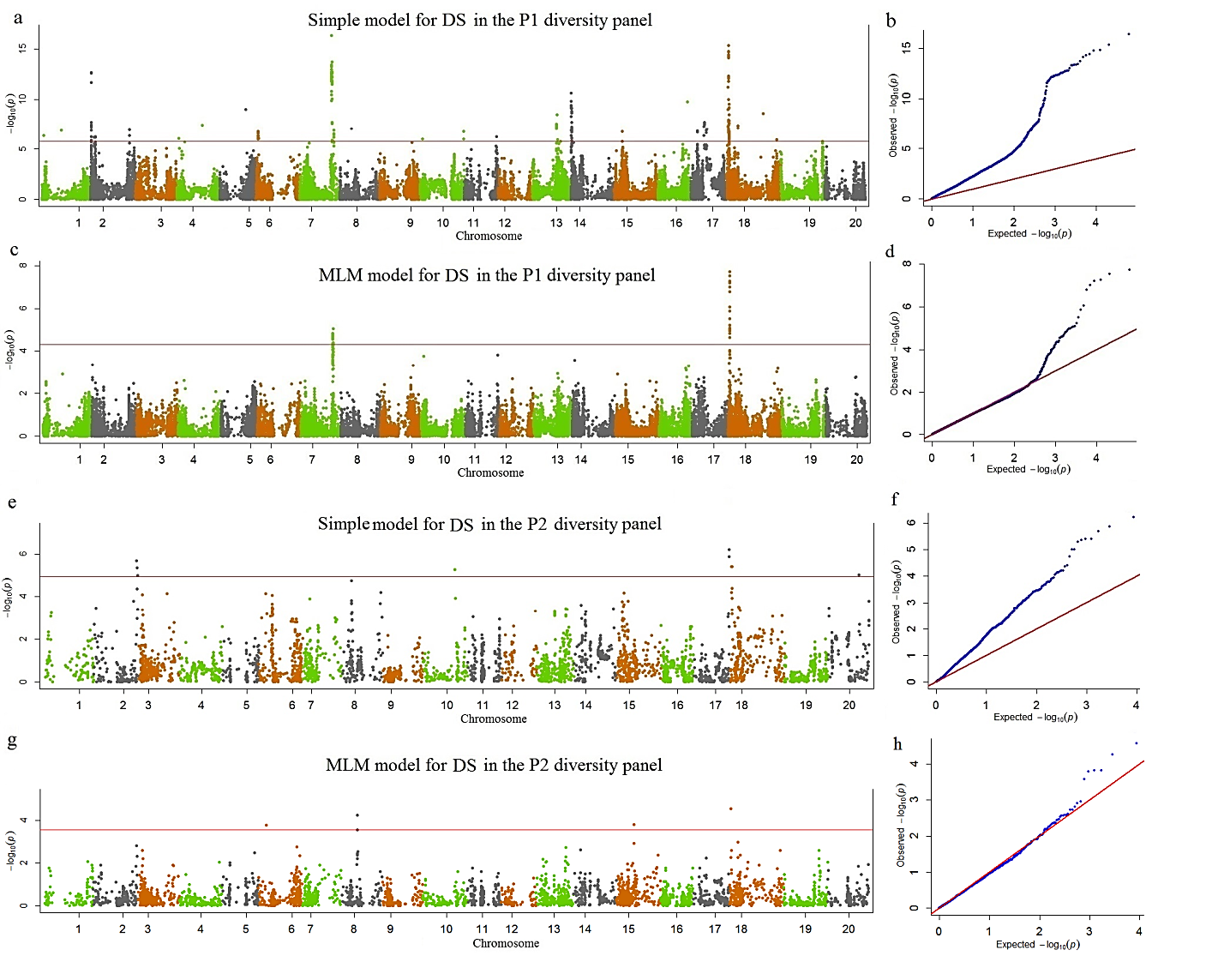
**

**Additional file 13** Genome-wide association study of DS in the two diversity panels. (**a**) Manhattan plots of the simple model for DS in P1 diversity panel. The −log10 *P* values from a genome-wide scan are plotted against the position on each of the 20 chromosomes. The horizontal red line indicates the genome-wide significance threshold (*FDR<0.05*). (**b**) Quantile-quantile plot of simple model for DS in the P1 diversity panel. (**c**) Manhattan plots of MLM for DX in P1 diversity panel, as in **a**. (**d**) Quantile-quantile plot of MLM for DS in P1 diversity panel. (e) Manhattan plots of the simple model for DS in P2 diversity panel, as in **a.** (**f**) Quantile-quantile plot of simple model for DS in the P2 diversity panel. (**g**) Manhattan plots of MLM for DS in P1 diversity panel, as in **a**. (**h**) Quantile-quantile plot of MLM for DS in P2 diversity panel.
