# Supplementary material for: Genome-wide association mapping of quantitative resistance to sudden death syndrome in soybean
Source: BMC Genomics. 2014 Sep 23;15(1):809. doi: 10.1186/1471-2164-15-809 (PMC4189206; doi:10.1186/1471-2164-15-809)
Supplement: Supplementary file 15 — Additional file 15: Regional plots showing association mapping results for SNPs located around Rfs2 / Rhg1 on chromosome 18. Negative log10-transformed P-values from the MLM are plotted on the left vertical axis for association panel P1; Negative log10-transformed P-values from the MLM are plotted on the right vertical axis for association panel P2. Blue horizontal dashed lines indicate the genome-wide significance threshold in association panel P1. Previously identified genes controlling the traits are labeled. (DOCX 94 KB) [file 12864_2014_6491_MOESM15_ESM.docx]

**
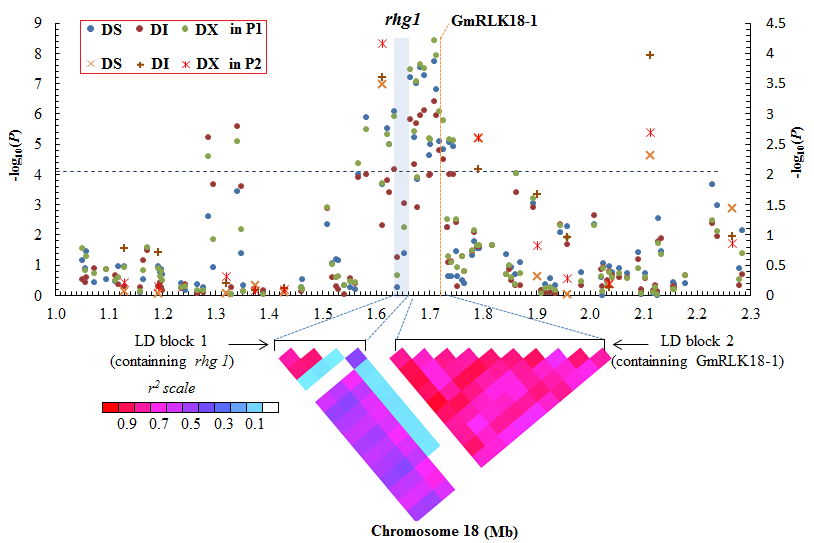
**

**Additional file 15.** Regional plots showing association mapping results for SNPs located around *Rfs2*/*Rhg1* on chromosome 18. Negative log10-transformed *P-*values from the MLM are plotted on the left vertical axis for panel P1; Negative log10-transformed *P-*values from the MLM are plotted on the right vertical axis for P2 association panel. Blue horizontal dashed lines indicate the genome-wide significance threshold in P1 association panel. Previously identified genes controlling the traits are labeled.
